# Synthesis, Characterization, and Sensor Applications of Spinel ZnCo_2_O_4_ Nanoparticles

**DOI:** 10.3390/s16122162

**Published:** 2016-12-17

**Authors:** Juan Pablo Morán-Lázaro, Florentino López-Urías, Emilio Muñoz-Sandoval, Oscar Blanco-Alonso, Marciano Sanchez-Tizapa, Alejandra Carreon-Alvarez, Héctor Guillén-Bonilla, María de la Luz Olvera-Amador, Alex Guillén-Bonilla, Verónica María Rodríguez-Betancourtt

**Affiliations:** 1 Department of Computer Science and Engineering, CUValles, University of Guadalajara, Ameca, Jalisco 46600, Mexico; alex.guillen@profesores.valles.udg.mx; 2 Advanced Materials Department, IPICYT, San Luis Potosí, S.L.P. 78216, Mexico; flo@ipicyt.edu.mx (F.L.-U.); ems@ipicyt.edu.mx (E.M.-S.); 3 Department of Physics, CUCEI, University of Guadalajara, Guadalajara, Jalisco 44410, Mexico; oscar.blanco@cucei.udg.mx; 4 Department of Natural and Exact Sciences, CUValles, University of Guadalajara, Ameca, Jalisco 46600, Mexico; msanchez@profesores.valles.udg.mx (M.S.-T.); alejandra.carreon@profesores.valles.udg.mx (A.C.-A.); 5 Department of Project Engineering, CUCEI, University of Guadalajara, Guadalajara, Jalisco 44410, Mexico; hguillenbonilla@gmail.com; 6 Department of Electrical Engineering (SEES), CINVESTAV-IPN, Mexico City, DF 07360, Mexico; molvera@cinvestav.mx; 7 Department of Chemistry, CUCEI, University of Guadalajara, Guadalajara, Jalisco 44410, Mexico; veronica.rodriguez@red.cucei.udg.mx

**Keywords:** spinel, faceted nanoparticle, cobaltite, sensors

## Abstract

Spinel ZnCo_2_O_4_ nanoparticles were synthesized by means of the microwave-assisted colloidal method. A solution containing ethanol, Co-nitrate, Zn-nitrate, and dodecylamine was stirred for 24 h and evaporated by a microwave oven. The resulting solid material was dried at 200 °C and subsequently calcined at 500 °C for 5 h. The samples were characterized by scanning electron microscopy (SEM), transmission electron microscopy (TEM), X-ray diffraction (XRD), and Raman spectroscopy, confirming the formation of spinel ZnCo_2_O_4_ nanoparticles with average sizes between 49 and 75 nm. It was found that the average particle size decreased when the dodecylamine concentration increased. Pellets containing ZnCo_2_O_4_ nanoparticles were fabricated and tested as sensors in carbon monoxide (CO) and propane (C_3_H_8_) gases at different concentrations and temperatures. Sensor performance tests revealed an extremely high response to 300 ppm of CO at an operating temperature of 200 °C.

## 1. Introduction

For a long time, pollutant gases from industry and internal combustion engines have been responsible for many human health issues, and to a large extent, for global climate change. Facing this, several groups have focused on the research and development of new sensor materials for the detection and monitoring of polluting gases such as CO and C_3_H_8_. In particular, sensor materials made from semiconductor oxides have been a valuable choice because they possess good chemical stability, low price, and an easy integration into electronic circuits. However, it is still necessary to improve sensor parameters such as selectivity, sensitivity, and operating temperature.

Zinc cobaltite (ZnCo_2_O_4_) is a p-type semiconductor material with a spinel-type structure. This material has attracted the attention of several research groups due to its potential applications as an electrode for Li-ion batteries [1,2,3,4,5], as a catalyst [6,7,8,9], and in supercapacitors [10,11,12,13]. In the gas sensors field, sensor devices based on ZnCo_2_O_4_ nanoparticles have displayed an excellent sensitivity to liquefied petroleum gas [14,15,16,17], H_2_S [18], ethanol [19,20], acetone [21], and formaldehyde [22,23], probably due to their high surface area. Additionally, ZnCo_2_O_4_ has also been used as Cl_2_, NO_2_, CO_2_, H_2_, NH_3_, CH_3_COOH, SO_2_, CO, C_3_H_8_, ethylene, xylene, toluene, and methanol gas sensors [15,16,18,19,20,21,22]. Specifically, when ZnCo_2_O_4_ nanoparticles were exposed to CO and C_3_H_8_ concentrations, a poor sensitivity was exhibited at working temperatures in the range of 175 to 350 °C [15,16,19,21]. In contrast, sensors based on nanowire-assembled hierarchical ZnCo_2_O_4_ microstructures showed a good sensitivity towards CO and C_3_H_8_ at an operating temperature of 300 °C [22]. From these studies, it is fair to assume that the gas sensing properties depend on the shape and size of the nanoparticles [24]. Therefore, developing a ZnCo_2_O_4_-based gas sensor, with a high sensitivity and a low operating temperature, is of great interest to many people. Several synthesis methods of ZnCo_2_O_4_ nanoparticles have been reported, such as combustion [1], hydrothermal [3,12], thermal decomposition [14], co-precipitation/digestion [15], water-in-oil (W/O) microemulsion [18], sol-gel [25], and surfactant-mediated [26] methods. In recent years, the microwave-assisted colloidal method has been an efficient and low-cost synthesis process for obtaining micro and nanostructures of oxide materials [27,28,29]. In fact, microwave is a simple technique, which plays an important role in colloidal synthesis since it provides a rapid evaporation of the precursor solvent and a short reaction time [30,31,32,33]. 

To the best of our knowledge, there are not reports on the synthesis and use of faceted ZnCo_2_O_4_ nanoparticles for gas sensing applications, such as the ones presented in this investigation, but in recent works, sensors based on faceted SnO_2_ and ZnSn(OH)_6_ nanoparticles exhibited high response towards toluene and ethanol, respectively [34,35]. We believe that materials based on faceted nanoparticles can be strong candidates for gas sensing applications due to their large surface areas. 

In this paper, ZnCo_2_O_4_ nanoparticles are synthesized via a microwave-assisted colloidal method [27,36]. The experimental method and the different steps to synthesize ZnCo_2_O_4_ nanoparticles are described in detail. In the following, the results of SEM, XRD, Raman, and TEM characterizations are shown. In addition, sensitivity results for sensors based on ZnCo_2_O_4_ nanoparticles are thoroughly discussed.

## 2. Materials and Methods

Zinc nitrate hydrate (Zn(NO_3_)_2_•xH_2_O, Sigma Aldrich 99.99%), cobalt(II) nitrate hexahydrate (Co(NO_3_)_2_•6H_2_O, Sigma Aldrich 98%), dodecylamine (C_12_H_27_N, Sigma Aldrich 98%), and ethanol (C_2_H_6_O, Golden Bell 98%) were used as reagents. 5 mmol (0.947 g) of Zn(NO_3_)_2_•xH_2_O, 10 mmol (2.91 g) of (Co(NO_3_)_2_•6H_2_O, and 5.4 mmol (1 g) of C_12_H_27_N were separately dissolved in 5 mL of ethanol. Additional syntheses were made with 10.8 mmol (2 g) and 16.2 mmol (3 g) of dodecylamine in order to obtain additional nanoparticle sizes. After 20 min of vigorous stirring on magnetic dishes at room temperature, the cobalt nitrate solution was added dropwise to the dodecylamine solution and kept stirring for 1 h. The zinc nitrate solution was then slowly added to the cobalt and dodecylamine mixture yielding a wine-color solution (the solutions with 10.8 and 16.2 mmol of dodecylamine were greenish-blue) with pH = 2 and a final volume of approximately 16 mL. This final solution was covered with a watch glass to avoid contamination and kept under stirring for 24 h, losing ~2 mL by evaporation. The solution (~14 mL) was evaporated afterwards using a microwave oven (General Electric JES769WK) at a low power (~140 W). The microwave radiation was applied over the solution for periods of 1 min in order to avoid splashing. The total time of evaporation was 3 h. The resulting solid was dried in air with a muffle (Novatech) at 200 °C. Finally, the calcination of the powder was done at 500 °C for 5 h in an alumina crucible with a cover at a heating rate of 100 °C/h. The sample was kept in the furnace for cooling at room temperature. The samples made with 5.4, 10.8, and 16.2 mmol of dodecylamine were labeled as A, B, and C, respectively. 

**Figure 1:**
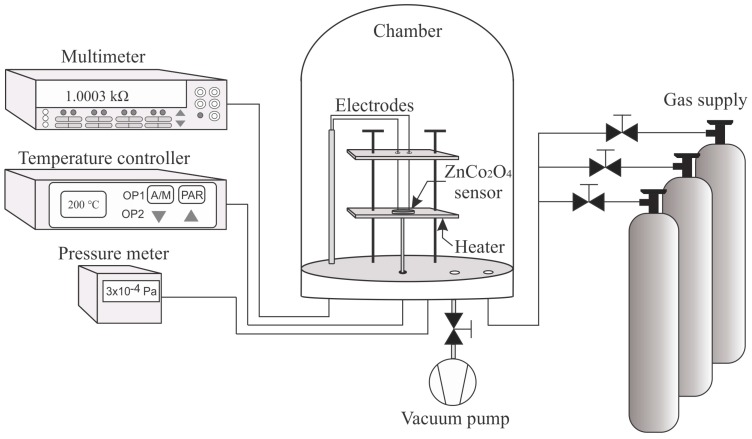
Experimental setup for the gas sensitivity measurements of ZnCo_2_O_4_ nanoparticles.

The calcined samples were characterized by SEM using a FEI-Helios Nanolab 600 system operated at 20 kV. XRD characterizations were performed with a PANalytical Empyrean system with CuKα and λ = 1.546 Å for phase identification. XRD patterns were obtained at room temperature in the range 2θ = 10–70° with steps of 0.02°, lasting 30 s for each step. The ZnCo_2_O_4_ crystallite size was calculated by Scherrer’s equation [37] using the plane (311) at 2θ = 36.8°:

(1) $$\mathrm{Cristallite}\;\mathrm{size}=\frac{0.89\lambda }{\beta \cos\theta }\frac{180{}^{\circ}}{\pi }$$

where λ is the X-ray wavelength and *β* is the full width at half maximum (FWHM). Raman spectroscopy characterization was performed using a Thermo Scientific DXR confocal Raman microscope with a 633 nm excitation source. The Raman spectra were measured from 150 to 800 cm^−1^ at room temperature, using an exposure time of 60 s and a Laser power of 5 mW. TEM, high-resolution transmission electron microscopy (HRTEM), energy-dispersive X-ray (EDS) and high-angle annular dark-field/scanning transmission electron microscopy (HAADF-STEM) were performed by means of a FEI Tecnai-F30 system operated at 300 kV. The gas sensing measurements were carried out on pellets of ZnCo_2_O_4_ with a thickness of 0.5 mm and a diameter of 12 mm. The pellets were prepared with 0.350 g of ZnCo_2_O_4_ powders employing a manual pressure machine (Simple Ital Equip) at 20 tons for 120 min. A TM20 Leybold detector was used to control the gas concentration and the partial pressure. The sensing response was investigated by measuring the electric resistance using a digital-multimeter (Keithley). The ZnCo_2_O_4_ pellets were exposed to several concentrations (1, 5, 50, 100, 200, and 300 ppm) of CO and C_3_H_8_. A schematic diagram of the gas sensing measurement system is shown in Figure 1. The gas sensing response, or sensitivity, was defined as S = R_a_/R_g_ for the reducing gas, where R_a_ and R_g_ refer to the resistances measured in air and gas, respectively [21,38,39].

## 3. Results and Discussion

**Figure 2:**
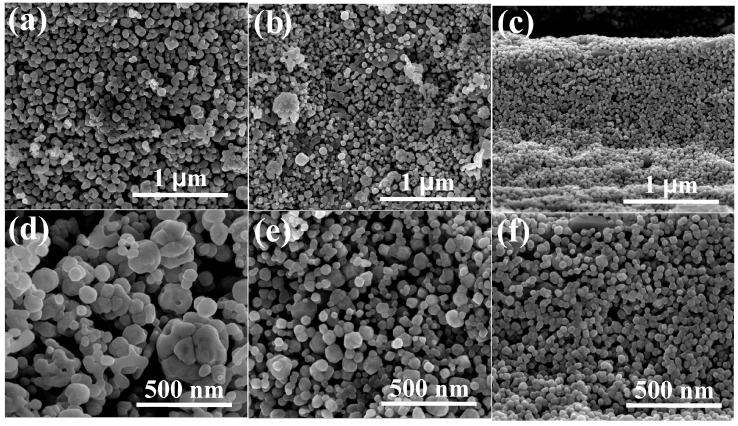
SEM images of ZnCo_2_O_4_ nanoparticles: (**a**,**d**) sample A; (**b**,**e**) sample B; and (**c**,**f**) sample C.

**Figure 3:**
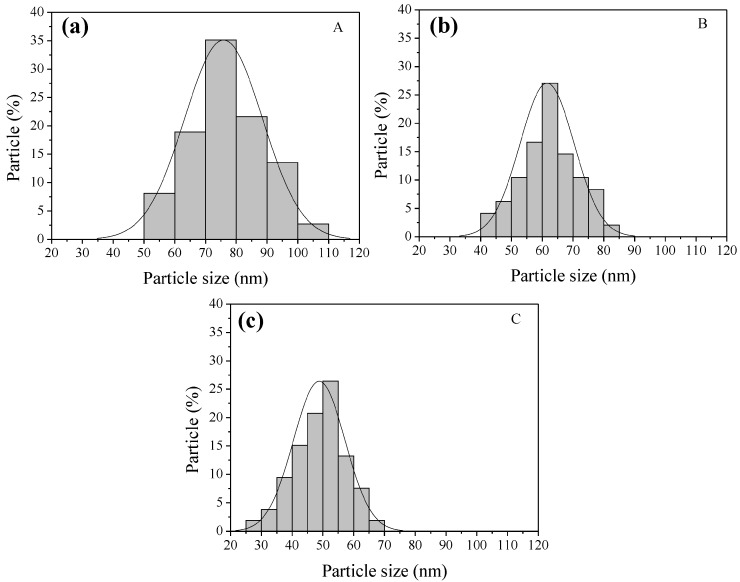
Particle size distribution for the ZnCo_2_O_4_ samples: (**a**) A; (**b**) B; and (**c**) C.

SEM images of samples A, B, and C are shown in Figure 2. Sample A exhibited a high concentration of nanoparticles with irregular shape and sizes in the range of 50–110 nm (see Figure 2a,d). Agglomerates of nanoparticles were also observed (Figure 2d). SEM images of sample B revealed a large amount of nanoparticles with diameters in the range from 40–85 nm (see Figure 2b,e). Sample C contained nanoparticles with diameters ranging from 25–70 nm (Figure 2c,d,f). The particle-size distribution of the three ZnCo_2_O_4_ samples can be seen in Figure 3. From SEM, it is clear that the dodecylamine concentration plays a key role in the morphologies and the particle sizes [40]: an increment of the dodecylamine concentration produced a large amount of nanoparticles and a decreasing particle size, which suggests that the dodecylamine inhibits the growth of the ZnCo_2_O_4_ nanoparticles. 

The formation of the ZnCo_2_O_4_ nanoparticles follows the principles of nucleation and growth established by LaMer and Dinegar [41]. In our synthesis, the nucleation process could occur when the zinc nitrate solution was added to the cobalt and dodecylamine solution [30], and the particle growth continued developing by diffusion of the nuclei during agitation of the final solution [42]. As previously discussed, dodecylamine plays an important role in the microstructure of the ZnCo_2_O_4_ particles. In this research, it seems that the ethanol and the dodecylamine are working synergically to reduce cations to metals because ethanol alone is not capable of doing so (i.e., in order to produce ultrafine metal nanoparticles), but just mixed with strong reducing agents such as microwave radiation, other chemical compounds, etc. [43,44,45]. We have observed that the dodecylamine concentration is the rate-determining factor; therefore, we propose the following reaction mechanism. 

Dodecylamine is a primary amine with properties of a weak base because of the nitrogen’s unshared electron pair. This kind of amine has nucleophilic behavior and can be used as reducing and surfactant agents. The reaction of dodecylamine with Co^2+^ cations could occur in two ways: (i) the attraction of the unshared electron pair to the nucleus of the cations in a nucleophilic reaction; (ii) the formation of an electrostatic bond between the nitrogen of the dodecylamine and the electrons of the outer shell of Co^2+^, because of the strong electronegativity of the nitrogen (= 3.04). The result of these interactions is the formation of the dodecylamine-Co^2+^ complex [46]:

(2) $${\mathrm{Co}}^{2+}+{\mathrm{NH}}_{2}{-(\mathrm{CH}}_{2}{)}_{11}{\mathrm{NH}}_{3}\to {\;\mathrm{Co}}^{2+}•••{\mathrm{NH}}_{2}{-(\mathrm{CH}}_{2}{)}_{11}{\mathrm{NH}}_{3}$$

In this reaction, dodecylamine is working as the surfactant; therefore, nitrogen’s electrons could participate as reducing agents, reducing the cationic Co^2+^ to metallic nanoparticles of Co^0^.

(3) $${\mathrm{Co}}^{2+}+2e^{-}\to {\;\mathrm{Co}}^{0}$$

For 5.4 mmol of dodecylamine, the molar ratio of dodecylamine: Co^2+^ was around 1:2, which suggests that half of the Co^2+^ cations are not participating in the formation of complexes and should be reduced in some way by the solvent. When the dodecylamine-ethanol-Co^2+^ solution is mixed with the ethanol-Zn^2+^ solution, and once the Co^2+^ and Zn^2+^ have been reduced, Zn could attract two atoms of Co to form ZnCo_2_, as Zn is slightly more electronegative than Co (1.90 vs. 1.65, respectively). Since 5.4 mmol of dodecylamine are not enough to complex all of the Co^2+^ cations, there are several available cations to be reduced by the solvent, and as a result the ZnCo_2_ grains are the larger ones. For 10.8 mmol of dodecylamine, most of the Co^2+^ should be forming a complex, and the growing of ZnCo_2_ grains is restricted through the slow release-reduction process of Co^2+^. For 16.2 mmol of dodecylamine, the Co^2+^ cations could be completely complexed; therefore, the excess of dodecylamine should form complexes even with Zn^2+^, restricting even more the growing of ZnCo_2_ grains. Summarizing, the role of dodecylamine is as a surfactant and as a reducing agent; however, it seems that the function of surfactant, through electrostatic interactions, is the dominant one, reducing the particle size and modifying the particles’ microstructure as the dodecylamine concentration increases. With heat treatment, the dodecylamine was finally removed from the ZnCo_2_O_4_.

**Figure 4:**
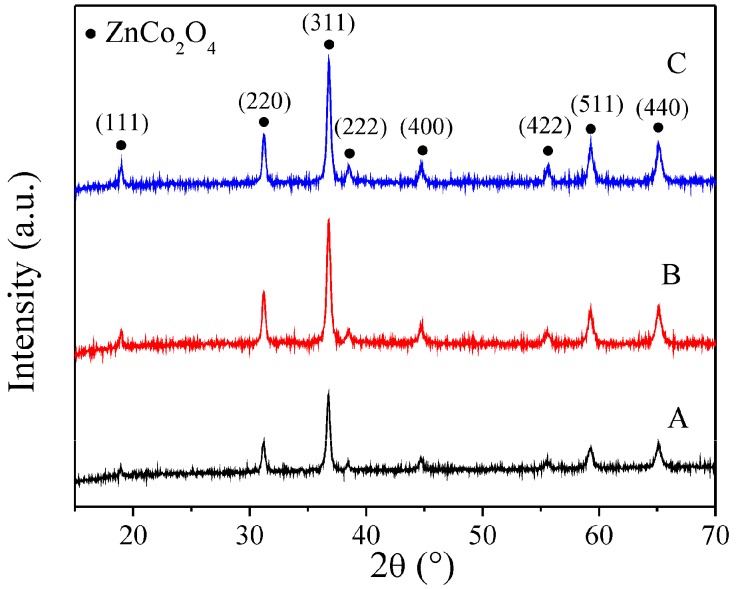
XRD patterns of ZnCo_2_O_4_ samples synthesized with (**A**) 5.4; (**B**) 10.8; and (**C**) 16.2 mmol of dodecylamine. The peak signals for the ZnCo_2_O_4_ structure (JCPDF 23-1390) are labeled with black-filled circles.

Figure 4 depicts the XRD patterns of the samples. In them, the typical peaks corresponding to the cubic ZnCo_2_O_4_ spinel-structure were identified. The well-defined narrow peaks are an indication of the good crystallinity of the ZnCo_2_O_4_ samples. Table 1 shows the crystallite size of every sample. From these results, an increase in crystallite size was produced when the dodecylamine concentration was increased. A slight increase in the FWHM was also observed, indicating a particle size reduction as was confirmed by SEM. 

**Figure 5:**
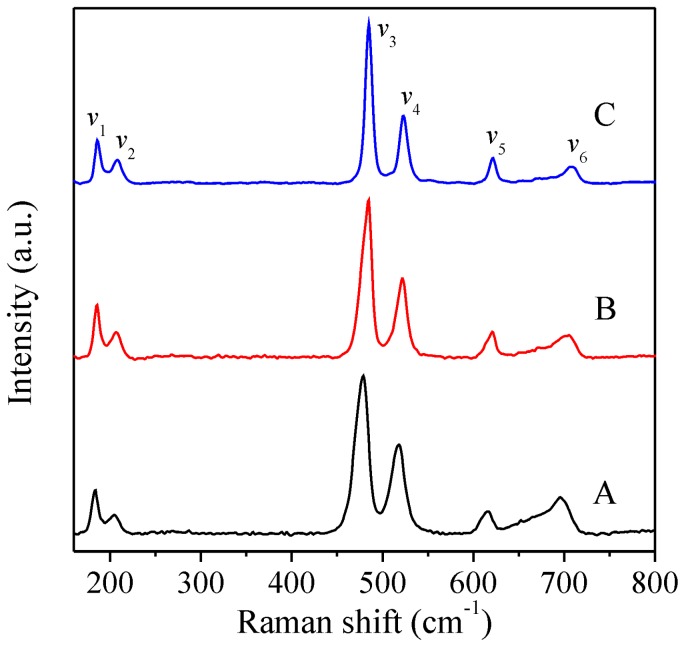
Raman spectra of the samples A, B, and C.

The formation of the oxide ZnCo_2_O_4_ was also confirmed by Raman characterization. According to the group theory for oxides with a spinel structure, five active Raman bands were expected: A_1g_ + E_g_ + 3F_2g_ [47]. Figure 5 shows the Raman spectrum indicating the main vibrational bands produced by the ZnCo_2_O_4_ spinel crystal structure. The bands labeled by *v*_1_, *v*_3_, *v*_4_, *v*_5_, and *v*_6_ correspond to 182, 475, 516, 613, and 693 cm^−1^, respectively. These bands are assigned to the F_2g_, E_g_, F_2g_, F_2g_, and A_1g_ symmetry species. These results are consistent with those reported for ZnCo_2_O_4_ spinel structures [48]. Interestingly, the band located at 204 cm^−1^ (*v*_2_) is a vibrational mode that could be attributed to a Co_3_O_4_ spinel structure [49]. The formation of Co_3_O_4_ is likely due to the cation disorder (substitution of Zn^2+^ by Co^2+^) in the spinel structure of the zinc cobaltite. Notice that the Co_3_O_4_ possesses a spinel structure similar to that of ZnCo_2_O_4_. Therefore, the XRD patterns between Co_3_O_4_ and ZnCo_2_O_4_ are almost indistinguishable. 

**Figure 6:**
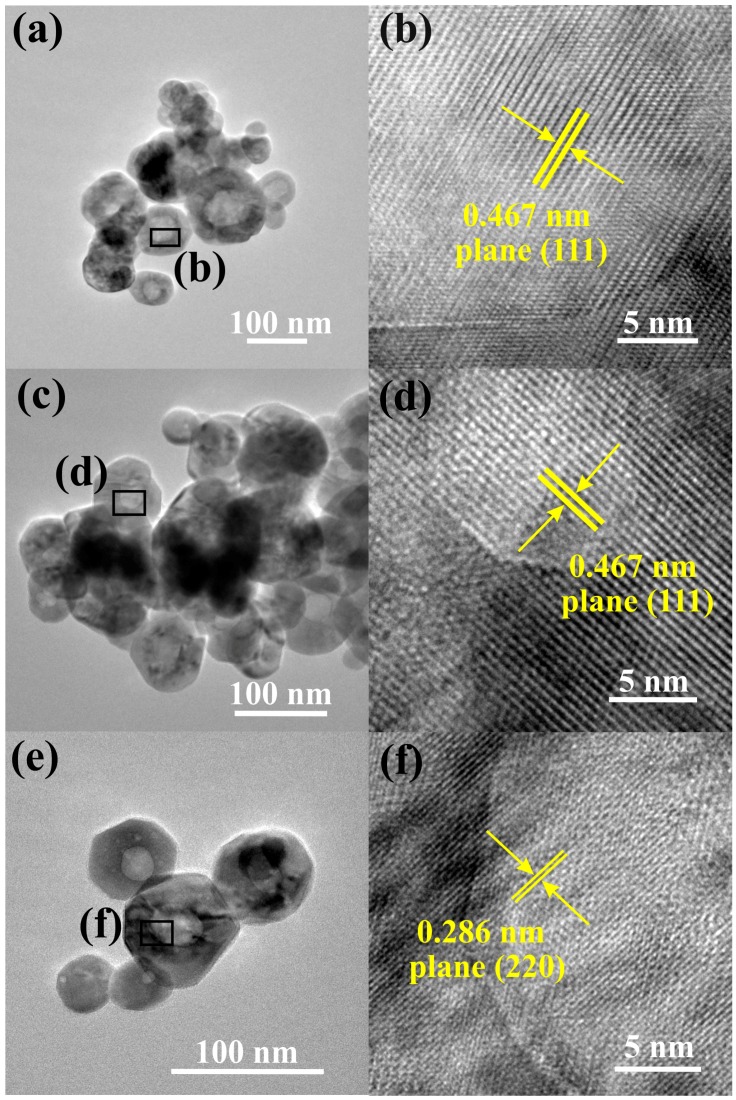
TEM and HRTEM images of the samples A (**a**,**b**); B (**c**,**d**); and C (**e**,**f**).

Figure 6 shows TEM images of the samples. The presence of faceted nanoparticles with a pockmarked structure was clearly identified (Figure 6a,c,e). The estimated average particle size was approximately 75, 61, and 49 nm for samples A, B, and C, respectively; these measurements are consistent with the SEM analysis. The standard deviation was ±12.64, ±8.78, and ±8.36 nm, respectively. Figure 6b,d,f shows HRTEM images of the selected area marked with a black rectangle. These images confirmed the presence of faceted ZnCo_2_O_4_ nanoparticles as was done by the XRD patterns and the Raman characterization. Fringe spacings of 0.467 and 0.286 nm are clearly observed, which are attributed to the planes (111) and (220) of the ZnCo_2_O_4_ spinel structure, respectively. 

**Figure 7:**
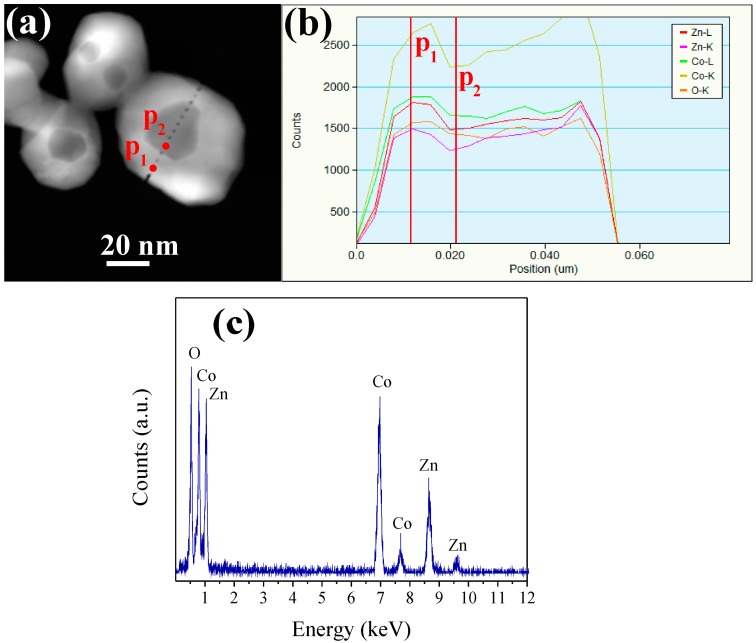
(**a**) HAADF-STEM image; (**b**) elemental line scan; and (**c**) EDS microanalysis of sample A.

To investigate the nanoparticle composition, an EDS line scan was performed on sample A (see Figure 7). Figure 7a shows a HAADF-STEM image of the ZnCo_2_O_4_ nanoparticles. The image confirms the presence of faceted nanoparticles with a pockmarked structure, which is consistent with the TEM images. In the EDS line scan, zinc, cobalt, and oxygen are observed across the linear mapping, confirming the presence of the expected elements, as seen in Figure 7b. In the central region p_2_, a decrease of the element composition is observed in comparison to point p_1_, which can be due to the irregular surface of the nanoparticle (pockmarked zone). Similar EDS elemental line scan spectra were obtained for samples B and C. Figure 7c shows an EDS microanalysis on p_2_, where the individual elements Zn, Co, and O can easily be seen. The atomic ratio of Zn:Co is approximately 1:1.96, which is consistent with the composition of ZnCo_2_O_4_. This EDS spectrum is in agreement with the literature [3,5]. 

**Figure 8:**
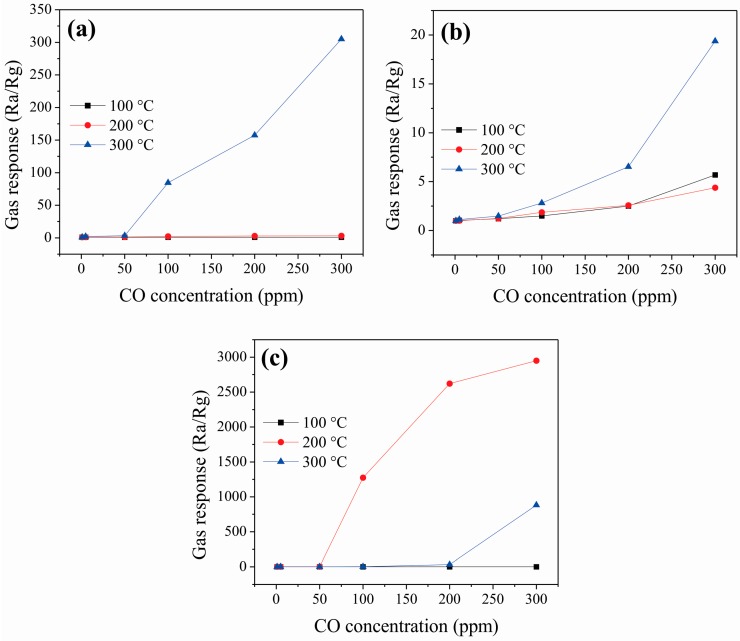
Gas response of ZnCo_2_O_4_ sensors vs. CO concentration at different operating temperatures: (**a**) sample A; (**b**) sample B; and (**c**) sample C.

To investigate the sensing properties of the ZnCo_2_O_4_ oxide, pellets of the material were made and tested in CO and C_3_H_8_ atmospheres. Figure 8 shows the oxide’s response vs. CO concentration of sensors made from samples A, B, and C. As shown in Figure 8a,c, no response variation was measured for A and C at 100 °C. On the contrary, the sensor made from B exhibited response values of 1, 1.03, 1.21, 1.48, 2.50, and 5.68 for CO concentrations of 1, 5, 50, 100, 200, and 300 ppm, respectively (Figure 8b). At 200 and 300 °C, the response of the three sensors increased with an increase of the CO concentration. In the whole concentration range (1–300 ppm CO), the sensor made from C exhibited a high response at 200 °C, better than the sensors based on A and B. At this temperature, the response values of the sensor based on C were 2.56, 2.66, 3.18, 1274.29, 2622.22, and 2950 for CO concentrations of 1, 5, 50, 100, 200, and 300 ppm, respectively. The sensors made from A and B comparatively also showed a good response to 300 ppm of CO (305.07 and 19.37, respectively) at 300 °C. From these results, it is clear that the ZnCo_2_O_4_ sensors are highly sensitive to concentrations of carbon monoxide and working temperatures. As expected, the material’s gas response increased due to the raising of the gas concentration and operation temperature. The raise of the response is associated to an increased oxygen desorption at high temperatures. Some authors report that the response of a semiconductor material depends on the adsorption of several oxygen species as a function of temperature [50,51]. The mechanism to explain the interaction between the CO molecules and a semiconductor oxide like the one used in this work is based on the accumulation layer's modulation due to the chemisorption of oxygen [33,52]. Therefore, in the tests at temperatures above 100 °C, the oxygen species O^−^ and O^2−^ (ionic form) that adsorb chemically on the sensor are more reactive than other oxygen species that adsorb at temperatures below 100 °C (like O_2_^−^) [29,36,37]. It means that below 100 °C, the thermal energy is not enough to produce the desorption reactions of the oxygen, and therefore, an electrical response does not occur regardless of the gas concentration. By contrast, above 100 °C (in this case, 200 and 300 °C), the formation of oxygen species occurs causing an increase in the gas-solid interaction in the presence of CO [33,52,53].

**Figure 9:**
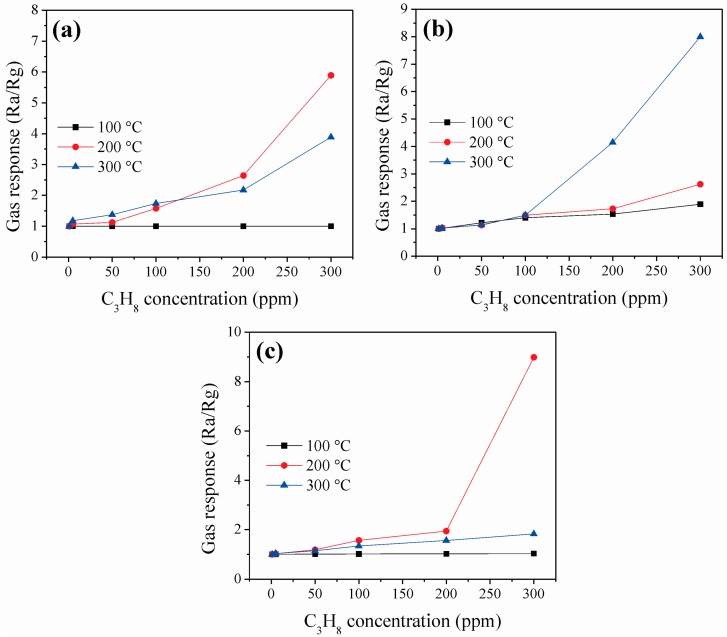
Response of the ZnCo_2_O_4_ sensors as a function of C_3_H_8_ concentration at different working temperatures: (**a**) sample A; (**b**) sample B; and (**c**) sample C.

The response of the ZnCo_2_O_4_ sensors in propane atmospheres at different operating temperatures is shown in Figure 9. Such as in the case of CO, the response rose with the increasing of temperature and propane concentration. However, at temperatures below 100 °C, no changes were observed in the response. At 100 °C, a sensing response value of ~1 was calculated for the sensors A and B in the range of 1–300 ppm of C_3_H_8_ (Figure 9a,c). At the same temperature, the sensor B registered values of 1, 1.03, 1.13, 1.49, 1.76, and 2.62 for C_3_H_8_ concentrations of 1, 5, 50, 100, 200, and 300 ppm, respectively (as seen in Figure 9b). Again, the sensor based on sample C also exhibited a higher response than those of A and B at a working temperature of 200 °C. The response values for this sensor C were 1, 1.03, 1.18, 1.56, 1.94, and 8.99 at C_3_H_8_ concentrations of 1, 5, 50, 100, 200, and 300 ppm, respectively. The sensors based on A and B also exhibited good response to 300 ppm of C_3_H_8_: 5.89 at 200 °C, and 8 at 300 °C, respectively. Additionally, the three ZnCo_2_O_4_ sensors showed a decrease in gas response when the test gases were removed from the vacuum chamber. 

As discussed above, the gas detection ability of a material such as the one used in this work depends on the microstructure obtained during the synthesis process [28,29,33,36]. If the particle size is nanometric, the response of the material is substantially improved [54]. It has been established that by reducing the particle size of the materials, their performance (i.e., their sensitivity) to detect different concentrations of gases is boosted [36,55,56,57], like in our case. Again, the most accepted mechanism to explain the response of the ZnCo_2_O_4_ is based on changes in the electrical resistance (or conductance) due to the adsorption and desorption of oxygen species on the surface [29,54,58,59,60]. Depending on the semiconductor type, the concentration of surface charge carriers can increase or decrease [59,61]. This is because during the chemical adsorption of oxygen molecules, a hole accumulation layer (space charge layer) is generated [52], provoking a chemical reaction between the gas and the surface of the ZnCo_2_O_4_ and resulting in changes in the electrical resistance of the material (i.e., a high sensitivity is recorded) [38,57]. Additionally, the ZnCo_2_O_4_ response is strongly related to the crystallite size (*D*), which is less than the thickness of the space charge layer, *L_s_*, defined as [29,54,55,59]:

(4) $$L_{s}=L_{D}\sqrt{\frac{e{V_{s}}^{2}}{kT}}$$

where *L_D_* is the Debye length, *e* is the electron charge, *V_s_* is the surface potential, *k* is the Boltzmann constant, and *T* is the absolute temperature. Generally speaking, *L_s_* has a value between 1 and 100 nm [56]. Therefore, the conductivity mechanism is associated with the crystallite size and the space charge layer [29,54,56,62]: if *D* >> 2*L_s_*, the conductivity is limited by the Schottky barrier at the particle border; thus, gas detection does not depend on the size of the particle; if *D* = 2*L_s_*, the conductivity and the gas sensing depend on the growing of necks formed by crystallites; and when *D* < 2*L_s_*, the conductivity depends on the size of the crystallites. In our case, the latter condition occurs while detecting the gases, since the average particle size is less than 100 nm; that is the reason why the conduction of the charge carriers (holes) takes place on the nanoparticles’ surface [56,63]. 

Comparing the efficiency of our ZnCo_2_O_4_ nanoparticles (a maximum sensitivity of ~2950 and ~8.99 in 300 ppm of CO and C_3_H_8_, respectively, at 200 °C both) with similar semiconductor oxides, we found in our case greater sensitivity, stability, and efficiency to detect CO and C_3_H_8_ at different temperatures. For example, references [51,64] reported that LaCoO_3_ has a maximum sensitivity in the range of 10 to 15 and approximately 42 for CO and C_3_H_8_ concentrations of 200 and 300 ppm, respectively, at 350 °C. For the SnO_2_ oxide, they reported a sensitivity of ~0.6 and ~0.7 at 300 °C in a concentration of 500 ppm for both gases. Reference [33] reported that the oxide ZnSb_2_O_6_ showed a maximum sensitivity of ~6.66 and ~1.2 at 250 °C for CO and C_3_H_8_, respectively. Reference [54] reported that the oxide CoSb_2_O_6_ had a sensitivity of ~4.8 at 350 °C in 300 ppm of C_3_H_8_. In the case of zinc-cobaltites, our ZnCo_2_O_4_ shows a superior gas sensing response than those synthesized by Vijayanand et al. [15], which had a sensitivity of ~1 in 50 ppm of CO at 350 °C; those by Zhou et al. (using sensors based on ZnO/ZnCo_2_O_4_ composites) [21], who obtained a response of ~1.1 in 100 ppm of CO at 275 °C; those by Liu et al. (using a sensor based on porous ZnCo_2_O_4_ nano/microspheres) [20], who obtained a response of ~2 in 100 ppm of CO at 175 °C; and recently, those by Long et al. [22], who reported a sensitivity of ~29 and ~13 at 300 °C in 10 ppm of CO and in 5000 ppm of C_3_H_8_, respectively.

## 4. Conclusions

In this work, we successfully synthesized ZnCo_2_O_4_ faceted nanoparticles with a size between 49 and 75 nm by means of a simple, economical, and efficient route: the microwave-assisted colloidal method using dodecylamine as a surfactant agent and a calcination temperature of 500 °C. Sensors prepared with these nanoparticles exhibited an excellent response (~2950 with sample C) at a relatively low operating temperature (200 °C) for the detection of CO, and they were capable of detecting up to 300 ppm of C_3_H_8_ at 200 °C, which is comparatively (with similar oxides) a very good performance. Hence, ZnCo_2_O_4_ is a promising material for applications as a gas sensor, especially in the detection of CO and C_3_H_8_.

## Figures and Tables

**Table 1:** Crystallite size of the ZnCo_2_O_4_ samples.

Samples	FWHM	Crystallite Size (nm)
A	0.338	24.75
B	0.351	23.84
C	0.421	19.92
